# Lessons from the first clinical trial of a non-licensed vaccine among Ugandan adolescents: a phase II field trial of the tuberculosis candidate vaccine, MVA85A

**DOI:** 10.12688/wellcomeopenres.14736.1

**Published:** 2018-09-19

**Authors:** Anne Wajja, Milly Namutebi, Barbara Apule, Gloria Oduru, Samuel Kiwanuka, Mirriam Akello, Beatrice Nassanga, Joyce Kabagenyi, Juma Mpiima, Samantha Vermaak, Alison Lawrie, Iman Satti, Jaco Verweij, Stephen Cose, Jonathan Levin, Pontiano Kaleebu, Edridah Tukahebwa, Helen McShane, Alison M. Elliott

**Affiliations:** 1 MRC/UVRI and LSHTM Uganda Research Unit, Entebbe, Uganda; 2 Vector Control Division, Ministry of Health of Uganda, Kampala, Uganda; 3 The Jenner Institute, Nuffield Department of Medicine, University of Oxford, Oxford, UK; 4 Laboratory for Medical Microbiology and Immunology & Laboratory for Clinical Pathology,, St. Elisabeth Hospital, Tilburg, The Netherlands; 5 Department of Clinical Research, London School of Hygiene & Tropical Medicine, London, UK; 6 School of Public Health, Faculty of Health Sciences, University of the Witwatersrand, Johannesburg, South Africa

**Keywords:** Tuberculosis, Vaccines, Clinical trials, Adolescents, Lessons, Challenges, Africa

## Abstract

**Background: **A more effective vaccine for tuberculosis (TB) is a global public health priority. Vaccines under development will always need evaluation in endemic settings, most of which have limited resources. Adolescents are an important target population for a new TB vaccine and for other vaccines which are relevant at school-age. However, in most endemic settings there is limited experience of trials of investigational products among adolescents, and adolescents are not routinely vaccinated.

**Methods: **We used *Modified vaccinia Ankara-expressing Ag85A *(MVA85A), a well-tolerated candidate vaccine for tuberculosis, to assess the effect of *Schistosoma mansoni *infection on vaccine immunogenicity among Ugandan adolescents in primary school. We describe here the challenges and lessons learned in designing and implementing this first clinical trial among Ugandan adolescents using a non-licensed vaccine.

**Results: **The school based immunization study was feasible and adhered to Good Clinical Practice principles.  Engagement with the community and all stakeholders was critical for successful implementation of the trial. Creative and adaptable strategies were used to address protocol-specific, operational and logistical challenges. This study provided lessons and solutions that can be applied to other trials among adolescents in similar settings elsewhere, and to school-based immunization programs.

**Conclusion: **Sufficient time and resources should be planned for community preparation and sensitization to ensure buy in and acceptance of a project of this kind. This trial shows that challenges to implementing early field trials in Africa are not insurmountable and that necessary well-planned high-quality ethical trials are feasible and should be encouraged.

**Trial Registration:** ClinicalTrials.gov NCT02178748 03/06/2014

## List of abbreviations

AIDS           Acquired Immune Deficiency Syndrome

BCG            Bacille Calmette-Guérin

CCA            Circulating Cathodic Antigen

ELISpot        Enzyme-Linked ImmunoSpot

ESAT-6        early secretory antigen target-6

CAB            community advisory board

CFP-10        culture filtrate protein-10

Ct                 Cycle threshold

EDCTP         European and Developing Countries Clinical Trials Partnership

HIV              Human Immunodeficiency Virus

HPV             Human Papilloma Virus

IFN-γ           interferon gamma

KK               Kato Katz

LCs              Local Councils

LSHTM         London School of Hygiene & Tropical Medicine

MOH            Ministry of Health

MRC            Medical Research Council

MVA85A *Modified vaccinia Ankara-expressing* Antigen 85A

NDA            National Drug Authority (NDA

PCR            Polymerase Chain Reaction

SATVI          South African TB Vaccine Initiative

*S.mansoni* *Schistosoma mansoni*

SOP             standard operating procedures

S.T.P.W.G.o.T.V     Stop TB Partnership Working Group on TB Vaccines

SSA             Sub Saharan African

TB               Tuberculosis

UNCST       Uganda National Council for Science and Technology

UVRI           Uganda Virus Research Institute

VCD            Vector Control Division

VHT            Village Health Teams

WHO          World Health Organization

## Background

Tuberculosis (TB) remains a global health problem with very high mortality and morbidity. In 2015, there were an estimated 10.4 million new TB cases ^1^. TB is one of the top 10 causes of death worldwide, and the leading cause of death from an infectious pathogen ^1^. The only licensed TB vaccine, Bacille Calmette-Guérin (BCG), prevents severe disease in childhood ^2, 3^ but does not protect against pulmonary TB among adolescents and adults in tropical latitudes ^4^. Development of a safe and effective TB vaccine is a key part of the World Health Organization (WHO) comprehensive strategy for combating the epidemic ^5^. An effective vaccine would revolutionise TB control ^6^. Candidate TB vaccines in development are designed either to replace the current BCG with more potent whole mycobacterial priming vaccines or to boost BCG given at birth. There are 15 new TB vaccine candidates and candidate combinations that are currently being evaluated in clinical trials ^5^. Despite the logistical and operational challenges, these vaccines will need evaluation in target populations in TB endemic countries where they are most likely to be deployed when licensed.

There is recent interest in, and need for, adolescent immunisation programs offering, for example, tetanus, hepatitis B boosting and Human Papilloma Virus (HPV) vaccination. Adolescents are therefore an increasingly important target population for school based immunization programs. In addition, adolescence is an important age group for booster TB vaccination ^7– 9^ because TB re-emerges as the pulmonary (transmissible) form in adolescents and young adults, after a relatively low incidence in childhood ^10^. Adolescents also harbour a high prevalence and intensity of helminth infection in countries where these are endemic ^11, 12^. It has been suggested that co-infection with helminths may contribute to the poor immunogenicity of vaccines seen in endemic countries ^13– 16^.

Despite the importance of immunization in this age group, and the relative ease of access via school, immunization in late primary school is not always routine in Sub Saharan African (SSA) countries. There are still many opportunities to learn from studies performed in this age group.

We utilised MVA85A, a candidate TB vaccine with an excellent safety profile ^17– 19^, as a model vaccine to evaluate the effect of infection with the helminth *Schistosoma mansoni* ( *S. mansoni*) on the T cell immunogenicity induced. Immunogenicity results showed that current *S. mansoni* infection had no effect on the vaccine-induced T cell response ^20^. This was the first clinical trial of an unlicensed vaccine in adolescents in Uganda. We share here experiences and lessons learned when implementing this trial. These are important for vaccine development in low income countries and for school based immunization programs.

## Methods

### Study design

This was a non-randomized, open label phase II clinical trial of MVA85A among adolescents with evidence of prior BCG vaccination, and with and without *S. mansoni* infection.

### Study setting and population

The study was conducted in primary school children on the shores of Lake Victoria in Wakiso district in Uganda. We selected schools with a high prevalence of *S. mansoni* based on previous surveys conducted by the Vector Control Division (VCD) of the Ministry of Health (MOH). VCD is mandated to monitor and treat helminths in schools and communities in Uganda, making this an important partnership for this project. Participants were recruited through their schools. We targeted children in upper primary classes. Preliminary visits and previous work by the VCD indicated there were sufficient numbers of 12–15 year olds in primary schools in the area. We initially selected two primary schools for the study but later added three more in order to achieve our target sample size. Enrolment challenges are discussed in the screening pool and selection criteria section.

### Ethical and regulatory approval

The study was reviewed and approved by three committees in Uganda – the Research and Ethics Committee of the Uganda Virus Research Institute, the Uganda National Council for Science and Technology (UNCST) and the Uganda National Drug Authority (NDA; which approves importation of licensed and unlicensed drugs) as well as ethical committees at Oxford University and the London School of Hygiene & Tropical Medicine in the United Kingdom.

The trial was registered with ClinicalTrials.gov under trial id NCT02178748 on the 03/06/2014.

### Pre-trial activities

Pre-trial activities included engagement with community leaders, district Ministry of Education and Health officials, school management and parents. We first presented and discussed the project with members of the district health service commission, which monitors all health-related matters in the district. Following this, we secured letters of approval from the district chief administrative officer on behalf of the Ministries of Health and Education introducing our team to the schools and community. We then met and discussed the trial with the head of the National TB Control Program since the study utilised a candidate vaccine for TB. At village level, we held meetings with members of the Village Health Teams (VHTs). VHTs are lay persons selected by the village and trained to provide or advise on primary or first-aid emergency care and other health related community initiatives. For example, VCD works with VHTs to distribute drugs during mass drug administration in the community, and mosquito nets. We held meetings with the chairpersons of the Local Councils (LCs) responsible for administration of the villages to make them aware of our presence and activities in their area of jurisdiction. The study area had a special population, a barracks housing prisons officers; consent for this population adhered to ethical guidelines for vulnerable groups in additional to the specific rules and requirements unique to them. For example, permission to access the barracks to provide information and to request parents or guardians to consent for their children’s participation in the study was restricted to particular times and required additional approvals from the head of the barracks.

We participated in parents’ meetings at each of the schools. Some of these were regularly-scheduled school meetings at which we were assigned a slot to present our work; others were organized solely to inform parents of our study. The project provided refreshments in the form of a soft drink at these parents’ meetings and offered travel reimbursement. The school introduced our team to the parents, and the team then explained the planned study to the parents as a group. We informed the parents of the study processes and that their children might be asked to participate following voluntary parental consent and child’s assent. We also addressed any questions raised.

### Pre-screening, screening and recruitment

First, from April to June 2014, all children in the five schools were pre-screened by the VCD as part of their routine mandate to monitor and treat helminths. This involved testing stool for helminths using the Kato Katz (KK) method. In addition to recording the child’s age and sex, the VCD team assisted us by recording the presence or absence of a BCG scar.

Next, formal screening for the trial commenced in June 2014. Participants were selected for screening based on pre-screening findings for age, gender, presence of BCG scar and Kato Katz results on infection status for *S. mansoni* and other helminths. Assenting adolescents, with consent from any one of the parents or guardian, were screened on the school premises.

At screening, participants provided urine, two further stool samples (on separate days) and blood. 

The initial laboratory investigations for screening were performed in the field by a laboratory technologist who was part of the trial team. These included tests for HIV, malaria and schistosome antigen (urine Circulating Cathodic Antigen (CCA)) using rapid diagnostic kits. If no exclusion criteria were met, samples were then transported in cold boxes approximately 20 kilometres along a busy highway from the school field site to laboratories within the Uganda Virus Research Institute (UVRI) campus in Entebbe. Efforts were made to transport the samples within two hours of collection. These were transported by the study vehicle, or by the field worker on a motorcycle when a faster delivery time was required. Sample tracking logs were used to monitor the process. Other screening tests included biochemistry, haematology, serology for hepatitis B and C, immunological assays and testing for helminths in stool by microscopy (Kato Katz) and PCR, detailed elsewhere ^20^. We used haematology reference ranges from a previous study conducted in Uganda, based on a healthy population, including adolescents ^21^.

We organized meetings with the parents and children to share results of the screening tests. At these meetings, we informed the parents individually of their child’s eligibility for the study and that we would be seeking their consent for their child’s participation. If ineligible for participation, the reasons for ineligibility were explained to both the parent and child. Ineligible participants were offered advice and/or referred to relevant facilities for treatment where needed.

Prior to vaccination, a further review was done to confirm participants’ eligibility including confirmation that both consent and assent had been given. The protocol allowed for 60 days between screening and vaccination. Eligible participants were enrolled and vaccinated on day 0 at the school premises. Enrolment was done consecutively at each school before moving to the next school. The vaccine was stored at -80°C in a secure, temperature-monitored freezer in the International AIDS Vaccine Initiative pharmacy located on the UVRI campus in Entebbe. It was transported to the field sites on dry ice on vaccination days.

Vaccination was done by the study team in the “temporary mobile clinic” set up on the school premises. The first participant was vaccinated on 11 ^th^ August 2014.

After vaccination, each participant was given a diary card to record any symptoms over a 6-day period post vaccination. They were each given a digital thermometer and a pen to take and record their temperature daily. A toll-free study-specific mobile phone line was kept with the study clinicians to advise on emergencies both during, and after, office hours. The number was available to participants, printed on the diary and study cards, and calls were received occasionally.

Vaccinated participants were again seen at the school premises on days (D) 7, 28, 42 (only for *S.mansoni* infected) and D56 post-vaccination. Stool samples for Kato Katz for all participants were performed at D28 and D56.

All participants were treated for helminths with praziquantel (40mg/kg) and albendazole (400 mg) under observation by trial staff. *S.mansoni* infected participants received two doses of praziquantel, the first on D28 (after samples were obtained) and the second one on D42, to optimise clearance of infection. Participants with no *S.mansoni* detected received a single dose on D56 (in accord with the annual mass treatment performed by VCD). All the pupils in the schools involved in this trial who were not enrolled in this trial were offered mass drug administration of praziquantel provided by VCD and administered with the help of staff at school using the validated standard dosing pole ^22^. The number of tablets given is based on the height of the participants.

### Inclusion and exclusion criteria

Participants were eligible for vaccination if aged 12 to 17 years, resident in the study area, BCG-vaccinated (based on BCG scar or written documentation) and healthy by history and physical examination. Exclusion criteria were clinical, radiological, or laboratory evidence of, or previous treatment for, active or latent TB (including a positive ELISpot for early secretory antigen target (ESAT-6) or culture filtrate protein-10 (CFP-10) at screening); sharing a residence with an individual on anti-TB treatment or with culture or smear-positive pulmonary TB within the last year; positive rapid diagnostic tests for HIV [(Alere Determine™ HIV–1/2 Ag/Ab Combo Catalogue No. 7D2649), (HIV 1 /2 STAT-PAK ^®^ DIPSTICK Assay HIV303 Catalogue No. 60-9534-0) and (Uni-GoldTM HIV Catalogue No. 1206502); positive serology for HIV (Murex Diasorin, Catalogue No. 9E25-02, HIV-1.2.0, Italy and Vironostika, Biomerieux, France), hepatitis B (surface Antigen Catalogue No. KMC 30011 and hepatitis C (Innotest HCV Ab IV Catalogue No. 80068(192T-CE), Innogenetics, Belgium); positive rapid diagnostic test for malaria (SD-Bioline Inc, Korea. Catalogue No. 05FK50); *Mansonella perstans* infection (modified Knott’s method); intestinal parasites other than *S. mansoni;* pregnancy; history of anaphylaxis or allergy likely to be exacerbated by vaccine; haematological or biochemical findings deemed clinically significant at screening and *S. mansoni* infection intensity of more than 2000 eggs per gram of stool (these individuals were treated immediately).

### Study endpoints

The primary endpoint was T cell immunogenicity assessed by *ex vivo* Ag85A-specific interferon gamma (IFN-γ) ELISpot response. Secondary endpoints included the profile of cytokine responses; plasma antibody concentrations to Ag85A, Schistosome Worm and Egg Antigens as well as any adverse events ^20^.

### Study results

Trial safety and immunogenicity data are described elsewhere ^20^. Here we focus on findings pertinent to the practical lessons learned.

A total of 1068 children were pre-screened by the VCD team at the five primary schools. Only 174 were within the target age group of 12–17 years and therefore approached for screening. Of these, 67 were excluded before consenting, 35 because parents/guardians did not consent; 12 had no BCG scar; 10 left school during the initial processes; five had no adult or responsible legal guardian accessible for consenting; three had recently been treated for schistosomiasis; one volunteer had known HIV-positive status; one was found to be under age at screening (11 years) despite having reported being 12 years at pre-screening. The main reason for many of the parents not consenting was unavailability due to work rather than any specific objection to the vaccine or participation in the trial.

A total of 107 participants who provided consent and assent were screened for eligibility. Only 36 (34%) of these were enrolled. A positive urine test for *Schistosoma* CCA, in spite of negative Kato Katz microscopy, among potential uninfected participants was the most common reason for screening failure (n=30, 42%). Other reasons included positive ELISpot at baseline indicating TB infection (n=19); positive rapid diagnostic test for malaria (n=10) and infection with other helminths (n=6). Five participants withdrew consent after screening. In three of the five instances where the reason for withdrawing consent was expressed, it was because one of the parents or the guardian (a grandfather and a brother) did not feel confident enough to take a decision for the child’s further participation in the study.

Each group had eighteen participants, although three participants in group 1, intended to be the no helminths group, had *S. mansoni* detected on PCR and were followed up to end of study and included in the safety analysis but excluded from the immunogenicity analysis. Infected and uninfected participants differed in age, school and hookworm ( *Necator americanus*) PCR status.

**Figure 1.**
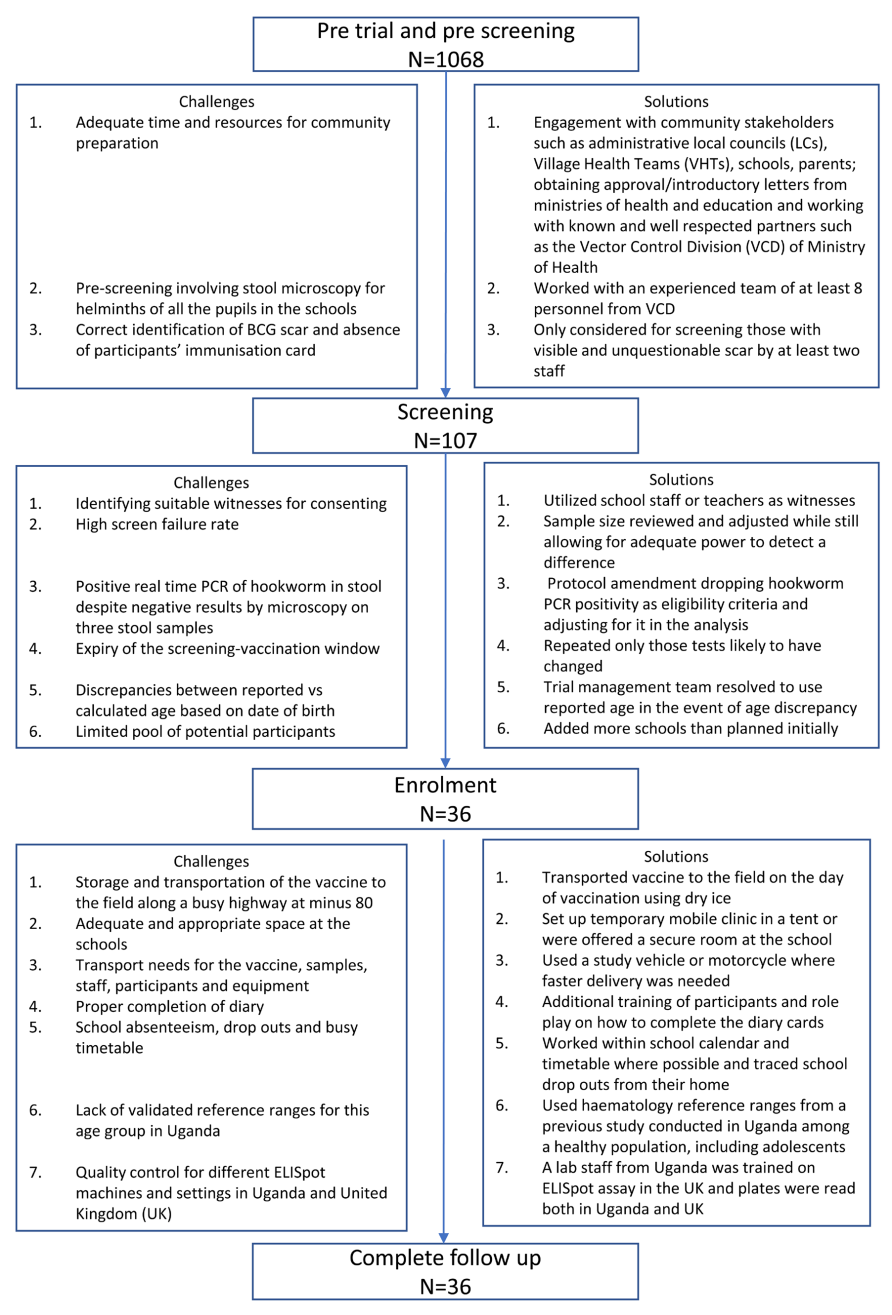
Summary of the challenges encountered and solutions during pre-screening, screening and follow up.

Figure 1 shows a summary of the challenges encountered and solutions during pre-screening, screening and follow up.

## Lessons learned and recommendations

### Engagement of stakeholders

The pre-trial meetings with community leaders, district Ministry of Education and Health officials, school management and parents effectively engaged stakeholders in the community and schools within the district and study area. These meetings were also a good opportunity for the team to tackle misbeliefs and rumours by providing correct information. For instance, in one of the schools, a couple of parents living in the same neighbourhood expressed a concern, based on rumours, that prior vaccination campaigns had led to several deaths in this community. Using information from the Ministry of Health on previous vaccination campaigns in that area, the team were able to confirm that this rumour was unfounded. This information was relayed to the parents in a subsequent meeting. It is important to make efforts to enhance two-way communication with communities to discuss issues that may be of concern to both the researchers and the community, and in so doing, help to close the vaccine confidence gap ^23– 26^.

Given the short time-frame (2-month duration of follow up), we did not establish a community advisory board (CAB), although these have become an integral part of studies with long follow-up periods. Community engagement activities, and CABs if needed, enhance community understanding of the research objectives and procedures, leading to mutual trust and a sense of collective ownership ^23, 27^. Findings from a study in rural Eastern Uganda ^28^ that explored the stakeholder perceptions about adolescent participation in a hypothetical TB vaccine trial in Ugandan adolescents indicated willingness to participate but highlighted the need for effective communication to the community with regards to the trial, its effects, and safety data, at an appropriate literacy level and in a readily understood format. In their study ^28^, adolescents and parents reported that their decision to participate in a trial would hinge on the quality of information provided, the effects of the vaccine, and other benefits such as access to healthcare and compensation; this concurs with a stakeholder perception study in South Africa, reported by Mahomed *et al* ^29^.

All our pre-trial activities and meetings were carried out alongside the VCD, which routinely works and delivers annual mass drug administration within this schistosomiasis endemic area and is well known and respected in the community. As reported by others ^24, 30^, collaborating with both government and non-government agencies already working on the ground and establishing relationships with key opinion leaders and stakeholders is essential.

As others have reported ^24^, the pre-trial activities and engagement were useful for community acceptance and smooth implementation of the project. The HPV pilot vaccination program in Uganda ^24^ showed that community buy-in could be achieved through a comprehensive understanding of information needs and effective communication strategies.

However, we did not minute our stakeholder meetings, or formally evaluate the impact and usefulness of these activities on the trial implementation and understanding of aims by the participants, parents and community. During a site visit, inspectors from the UNCST commented that more documentation of these meetings, such as the questions and concerns raised by the parents, and whether they understood what was presented, would have been useful. Innovative methods of engaging adolescents and the community have been used by other researchers enrolling adolescents in TB vaccine trials. The South African TB Vaccine Initiative (SATVI) used drama to engage and raise awareness of TB and clinical trials among adolescents ^25^. They followed through by assessing the impact of the drama on the adolescents. We therefore recommend that sufficient time and resources should be planned for community preparation and sensitization to ensure community buy in and acceptance of a project of this kind, and that engagement activities are appropriately monitored and evaluated to reassure regulators and to inform future work.

### Screening pool and selection criteria

The schools provided us with initial estimates, based on age, of the pool of potential participants. These turned out to be inaccurate and an overestimate. For example, one of the schools indicated that they had over 100 children in the upper primary classes (our target age group) but we found only 59 during pre-screening. We therefore quickly exhausted the pool during screening and had to alter our initial recruitment plan from two to five schools. The figures were usually based on the number of school attendees who had sat exams in the previous year, which was not always an accurate reflection of average attendance during the regular school period. Many children stay away from school doing household chores and other family activities such as farming, stone quarrying and fishing, and only show up later in the school term and closer to the examination period. Working closer to end of term rather than earlier in the school term yielded better numbers, but it was important to work with minimal interruption to the students especially during examination periods. It is important to be aware of such issues that may affect school attendance and plan accordingly.

We noted to our surprise a very high percentage of hookworm PCR positivity even for those participants that had three samples negative for hookworm on microscopy. These however had a high cycle threshold (Ct) value which is indicative of light infections that could have been hard to detect using the Kato Katz method. As described by others ^31^, this real-time PCR method is more sensitive than the conventional microscopic detection of hookworm infections. We had to amend the protocol to remove hookworm PCR positivity from the exclusion criteria because it would have been impossible to recruit the required number of children with *S. mansoni* infection and a negative hookworm PCR. We opted to adjust for hookworm PCR status in the analysis.

As reported by other African research sites ^21, 32, 33^, we did not have locally applicable ‘normal’ reference ranges for laboratory tests of this adolescent population. Published reference ranges are usually based on Western populations. It was agreed during protocol development to use reference ranges for haematology that catered for adolescents from a previous study ^21^ and to discuss abnormal screening biochemistry results in the trial management team meetings.

Our high exclusion rate meant that we encountered sample size challenges, and it was not possible to extend recruitment due to funding constraints. The original sample size proposed was 40–60 infected and 30 uninfected volunteers. We revisited the power calculations and found that enrolling approximately 12–24 children per group would still give us sufficient power (82% to 94%) to detect a two-fold reduction in immunogenicity in the *S. mansoni* infected group ^20^. A two-fold difference in immunogenicity is a minimum level of clinical and immunological significance. Our final sample size of 18 in each group was a pragmatic compromise between desired study power and logistical challenges. Whereas we acknowledge the possibility that small but potentially interesting effects may have been missed, there was no suggestion of an effect of helminth infection on vaccine immunogenicity ^20^.

Given the anticipated gender and age differences in the prevalence of helminths, the initial plan was to group-match by age and gender at enrolment within 6 strata (males aged 12-13, males aged 14-15, males aged 16-17 and females aged 12-13, females aged 14-15 and females aged 16-17). This proved a challenge to recruitment and a further protocol amendment was necessary to reduce this requirement to controlling for age and gender in the analysis, instead of age stratification.

Careful consideration of protocol challenges and development of well thought out amendments during the trial allowed us to adapt and respond to recruitment challenges while ensuring that the study remained scientifically sound.

### Operational and logistical issues

Early-phase clinical trials are often carried out in purpose built clinics but this may not be appropriate or feasible when conducting field studies on endemic and neglected infectious diseases in the target population. For this field trial, on vaccination days, the vaccine was transported on dry ice to the field sites located about 20 kilometres along a busy and usually traffic-congested highway. The field team went to the field site first to prepare the participants, and communicated to the driver and internal monitor to bring the vaccine once the team was ready. This was a logistical challenge which was resolved with good communication and coordination within the team.

Space at the field sites was another challenge given that schools are not designed to have such activities. Necessary equipment included resuscitation tools and oxygen, and arrangements for emergency care were made with nearby hospitals. Where available, the school administration provided space or a room for study activities and secure storage for equipment and supplies. If unavailable, the team moved with these items on a daily basis. Given the set up, we did not always have a clean and private space for counselling and procedures but improvised using a tent and movable screens. We hired a local resident to work as a security guard whenever we left the erected tent onsite during the weekend.

The study had transportation needs for the vaccine, samples, staff, participants and equipment. We relied heavily on the single study vehicle and the field worker’s motorcycle and occasionally hired additional transport services when needed.

We therefore recommend that there is careful consideration of field provisions for privacy, point of care lab testing and handling of samples, provision of investigational products, equipment transport and security arrangements for study equipment and confidentiality of documents.

### School specific issues

Our study required working with adolescents aged 12 to 17 years attending school. It was important to schedule our activities in parallel with the school calendar and timetable. This included planning for school holidays, examination periods, school meetings and sending letters of invitation to the parents. We tried to avoid interruption of lessons by working within the pupils’ break periods. At the onset, we also planned not to recruit participants in candidate classes for national Primary Leaving Certificate Examinations so as to avoid disrupting their studies, especially during the final examination period. Enrolled participants were reminded the day before of their next visit and in some instances collected from home by the study driver or field worker on the visit day if they had missed school. Some of the children who participated in the pre-screening and screening activities had dropped out of school by the time we returned to enrol them. The school administration, teachers, parents and children worked well together. Several teachers served as witnesses for illiterate parents during the consenting process. However, at times this encroached on their teaching responsibilities and had to be managed effectively. We emphasized to the parents that consent was voluntary and there were no consequences for declining to consent.

Other studies ^26, 34^ in Africa have reported similar challenges including school absenteeism and incomplete records. This can be more common during specific seasons such as rainy season for farming or fishing. Community strategies to address school absenteeism, such as encouraging attendance by providing meals, may help ^26, 34^. Our study paid for the school meals for the whole school on the day mass drug administration was given specifically to target side effects related to use of praziquantel on an empty stomach ^35^.

At the school level, collaboration with the school administration and management was key for successful implementation of the project. Careful planning in consideration of the school calendar and activities would be necessary for successful implementation of a similar school-based immunization program. Our study indicates that school based immunization programs can be made possible with minimal interruption to school activities. They also provide an opportunity for integrated healthcare services for adolescents. Our study activities were tagged to the mass drug administration to schools in schistosomiasis endemic areas done by VCD. Others have incorporated screening for alcohol and tobacco use in South Africa ^36^; deworming and distribution of Vitamin A in Uganda ^34^ and health promotion in Rwanda ^37^. Combining HPV vaccine delivery with other adolescent programs ^24^ was an efficient strategy for good use of limited resources.

### Ethical and regulatory issues

The review process involved three ethics committees and two regulatory bodies (see ethical and regulatory approval) and took seven months in total. This was the first vaccine trial of a non-licensed product among the vulnerable adolescent population in Uganda, and the NDA sourced external reviewers, as well as in-house reviewers, for this content area. A meeting to explain further the study to the NDA and address queries was helpful to obtain the final approval. 

It is important to anticipate such delays, especially for studies in vulnerable populations. Where acceptable, concurrent submissions can expedite the process. Extra efforts to present as much information as possible and face to face meetings can help, particularly for new study areas or populations. Several institutions and funding agencies, such as the European and Developing Countries Clinical Trials Partnership (EDCTP) and WHO, are supporting the strengthening of both ethical committees and regulatory authorities in Africa ^38^.

The trial had an in-house internal monitor who was also delegated by the sponsor to serve as the local safety monitor. External monitoring, meeting the expected international standards, was performed by the EDCTP-funded reciprocal monitoring scheme at a reasonably affordable cost. UNCST selected this study for a supervisory site visit because they were especially interested for purposes of benchmarking the specific use of a new investigational product in the adolescent population. They were satisfied with its conduct and documentation.

Each participant had to have both assent and consent to be enrolled. Assenting was mostly performed at school while consenting was carried out either at school or at home. In anticipation of the challenges, it was agreed up-front in the standard operating procedures (SOPs) that assent and consent did not have to be done simultaneously on the same day or time. The children provided assent independently of their parents. For the consent process, identification of suitable witnesses, where needed, was easier at school but at times posed a challenge at home.

### Protocol and study specific issues

Given the many screening tests, and challenges in tracing some participants, the 60-day screening window expired before enrolment for a few participants. A decision on how to proceed on these issues was discussed and agreed during the weekly trial management team meetings on a case-by-case basis by repeating only those screening tests for which results might have changed beyond the accepted screening window period.

The field trial team was small: a project leader who is a medical doctor, a clinical officer, a nurse, a field laboratory technologist and a field worker. Each member of the team had a back-up identified and trained from within the wider host research programme at the start of the trial, although these were equally busy at their primary station and therefore not readily available. All trial team members were trained and delegated to perform the general roles such as consenting and assenting. This helped to ensure better work flow in the event that a team member was unavailable. Staffing challenges are reported in other studies ^39^ and creative strategies to address them should be devised.

In our setting, most people do not have birth certificates or records but report a particular age based on what their parents told them. When asked during pre-screening and screening, many children reported a slightly different age from the one calculated from their reported date of birth when completing the questionnaires. In such circumstances, the trial management team agreed to use the reported age. Mugisha *et al.* ^34^ found similar challenges and reported that determining age eligibility was easier using a grade-based strategy that relied on child’s school grade/class rather than an age based strategy which attempts recruitment based on age. Similarly, the pilot HPV vaccination program in Uganda ^24^ reported that it was easier to implement school based immunization programs based on grade/class in school, rather than age. Whereas this may be a practical solution during implementation of programs, it may not be acceptable for regulations and guidelines for clinical trials.

Most of our participants did not have their child health immunization card or record. For evidence of BCG vaccination, we relied on presence of BCG scar which may have excluded participants who were vaccinated but did not have a clearly visible scar.

Our study participants initially experienced some difficulties in completion of diary cards at home, with some checking off all symptoms and all severity grading. More emphasis on training the participants on how to complete the diary cards was done subsequently prior to vaccination. This required more time but was beneficial in the long run.

## Conclusion

We have described the challenges and lessons learned in designing and implementing this first clinical trial among Ugandan adolescents using a non-licensed vaccine. This study provided lessons that can be applied to other trials among adolescents in similar settings, and to school-based immunization programs. The solutions we implemented will be of value in planning other trials in this vulnerable population in Uganda and in similar settings elsewhere. This trial shows the challenges in implementing early-Phase field trials in Africa are not insurmountable and well planned ethical trials are feasible and should be encouraged.

## Declarations

### Ethics approval, consent and assent to participate

The study was reviewed and approved by three committees in Uganda – the Research and Ethics Committee of the Uganda Virus Research Institute [Reference GC/127/14/01/444], the UgandaNational Council for Science and Technology (UNCST) [Reference HS 1565] and the Uganda National Drug Authority (NDA; which approves importation of licensed and unlicensed drugs) [Reference 198/ESR/NDA/DID-05/2014], as well as the Oxford Tropical Research Ethics Committee at the University of Oxford [Reference 1060-13] and the Observational/Interventions Research Ethics Committee at the London School of Hygiene & Tropical Medicine in the United Kingdom [Reference 7283]. Informed consent for the adolescents to participate in the trial was sought from their parents or guardians. Assent to participate was sought from the adolescents.

## Data availability

The main results of this trial with full methods have been reported previously: https://doi.org/10.1371/journal.pntd.0005440 ^20^

The datasets generated and/or analysed during the study are available without a license in the Jenner Institute, University of Oxford repository: Article ID PNTD-D-16-01618R1 http://www.jenner.ac.uk/pntd-d-16-01618r1
