# Supplementary material for: Lessons from the first clinical trial of a non-licensed vaccine among Ugandan adolescents: a phase II field trial of the tuberculosis candidate vaccine, MVA85A
Source: Wellcome Open Res. 2018 Sep 19;3:121. [Version 1] doi: 10.12688/wellcomeopenres.14736.1 (PMC6338128; doi:10.12688/wellcomeopenres.14736.1)
Supplement: Peer review history

# Peer review: Referee response for version 1

**DOI:** 10.21956/wellcomeopenres.16054.r34468

**Published:** 2019-01-11

**Authors:** Dessislava V Marinova

**Affiliations:** 1 Department of Microbiology, Faculty of Medicine, University of Zaragoza, Zaragoza, Spain

The manuscript by Wajja Anne *et *al., sent for publication as a research article in Wellcome Open Research Journal focuses on the lessons learned from the technical, operational and logistic challenges encountered upon conducting an open label Phase II field trial with the tuberculosis vaccine candidate MVA85A, a model candidate tuberculosis vaccine, to assess the effect of helminth *Schistosoma mansoni* ( *S. mansoni*) infection on vaccine-induced immunogenicity (and safety) in BCG-vaccinated Ugandan adolescents.

 Authors describe that this was the first clinical trial(school-based immunization study) of an unlicensed vaccine in adolescents in Uganda and show that pre-trial engagement with the community and all stakeholders, including community leaders, district Ministry of Education and Health officials, school management and parents is critical for successful implementation of a project of this kind.

 This work provides lessons and solutions that can be of great value for the design and implementation of clinical trials among adolescents in similar settings, including TB vaccine clinical trials targeting specifically this age group population where incidence of pulmonary (transmissible) forms of TB is considered very high, as well as to school-based immunization programs. This article presents scientifically sound work suitable for publication in Wellcome Open Research Journal.

 Comments for the authors

  

1. **Abstract and Background in main text**: Although the scope of the article is the description of challenges and solutions implemented for the successful conduct of this first clinical trial in Ugandan adolescents, the specific rationale of the clinical study and relevance of the study results is not clear. Briefly describe study rationale and how results can serve the scientific community in these two sections.

 

1. Since this study is very useful for implementing new clinical trials in adolescents in Uganda and in similar settings, it would be useful that authors comment on how it was dealt with convincing the community and stakeholders on using a candidate TB vaccine which has been shown to be ineffective against tuberculosis for the purposes of the current study.

I have read this submission. I believe that I have an appropriate level of expertise to confirm that it is of an acceptable scientific standard.

# Peer review: Referee response for version 1

**DOI:** 10.21956/wellcomeopenres.16054.r34469

**Published:** 2019-01-04

**Authors:** Benjamin M Kagina

**Affiliations:** 1 Vaccines for Africa Initiative, University of Cape Town, Cape Town, South Africa

In this manuscript, the authors document the challenges experienced and lessons learnt while conducting a phase II trial of a non-licensed TB vaccine (MVA85A) among Ugandan adolescents. The authors have shared some practical steps taken by the study teams to address the challenges encountered during the clinical trial. 

 The authors have contributed useful knowledge (specifically on the operational aspects) in the field of adolescent immunisation. Research on adolescent immunisation, whether primary (with vaccines such as HPV) or booster (e.g Tdap) or catch up (e.g measles) or testing vaccines (e.g TB vaccines) is limited. Therefore, the authors must be commended for this relevant scientific contributions to the field. 

 Here are some comments for the authors to consider:

 1) Abstract and background- because this study was nested within a clinical trial, clarity of the current study rationale and methods is lost at the expense of the main study (clinical trial). Emphasize must be given to the rationale and methods that are relevant to the findings of this manuscript.

 2) Study endpoints: this section is completely misaligned with the study findings and this affects clarity.

 3) Methods/results/discussion- there are several known individual related factors that can affect adolescent vaccination (or engaging this population to participate in clinical trials). One of the factors is lack of optimal knowledge on immunisation, vaccines and VPDs by the adolescents themselves, teachers as well as their parents. If the authors never specifically investigated this factor, discussions on this and other related factors would be useful. 

 4) Authors should mention if these results can be extrapolated to similar settings in Africa. Are there study limitations? Related to the study limitations, what would be the most optimal study design to investigate the challenges discussed in this study? This information can be the entry point of future follow up studies.

 Minor comment: be consistent when using the term "school-based". Sometimes the term has an hyphen and other times, the term is not hyphenated.

I have read this submission. I believe that I have an appropriate level of expertise to confirm that it is of an acceptable scientific standard, however I have significant reservations, as outlined above.

# Peer review: Referee response for version 1

**DOI:** 10.21956/wellcomeopenres.16054.r33979

**Published:** 2018-11-06

**Authors:** Neil French

**Affiliations:** 1 Institute of Infection and Global Health, University of Liverpool, Liverpool, UK

The paper sets out to describe the lessons learned from performing a trial of an experimental TB vaccine in adolescents. I was anticipating a qualitative or semi-quantitative evaluation of key issues relating to recruitment and follow up of this population group, but the paper contained a more subjective description of activities, including several generic issues which are not unique to this particular study. I enjoyed the read but was not left with the feeling that the there was a clear set of recommendations that will enhance the prospects of researchers who are new to this area. The authors should consider how they might focus this down to a more specific critique of the issues for adolescent/school studies. As a minimum they should distinguish between generic clinical trial issue and those features that were specific to or interactive with this group of adolescents in all parts of the document.

 Introduction: this describes the nature of the study and the rationale for undertaking a study in adolescents of this vaccine. It did not contain any reflection on clinical trials undertaken in adolescents as a whole. It would have been valuable to have some statement of what has been done in this area to date.

 Methods: this describes the study recruitment intervention and follow up. This study has been published elsewhere, so the methods could have been made more concise by focusing down on specific areas that are unique to undertaking a trial in adolescents e.g. less on samples taken and more on what parts of the pre trial discussions were specific to this trial and would not be part of an adult trial. I suspect discussion with the  district health commission is something that goes on with every study, adding information to show what was unique in the methods would be very helpful. A little more information on the schools would also be helpful – size, number of teachers, catchment areas. Were these all government funded schools?

 Results: the breakdown of screening and recruits and follow up by numbers and reasons is valuable. The last paragraph that indicates the status of the final recruits by helminth status is a little distracting as this has probably been reported in the main paper and doesn’t add a great deal to the paper. Were there any follow up issues?

 Lessons learnt: The pre trial meetings, better description of what was specific is needed, either here or in methods. A comment is made about sufficient time and resources, could this be quantitated in some way – how many meetings, how many people involved, how much time was invested in this. What I would also like to know was if there were any issues raised by the various groups that were related to the study group.

 The VCD team what specific actions did they undertake that progressed the study or was this just familiarity? Could this have worked in a negative way – this group were trusted which limited critical community review?

 In figure 1 I found the mixing of generic study issues with target group specific issues unhelpful. For instance in the enrolment section challenges box issues such as machine validation and transport needs are generic issues for trials in low income settings, whereas reference ranges and school absenteeism are specific and seem of greater relevance to this paper. As a minimum please separate or sub head these. I am surprised you didn’t insert details in this table about potential recruitment assessment as this was a challenge, in so much as the figures were not accurate. Why was a secondary school not chosen for this study – this could be described in greater detail.

 The paragraph on PCR positivity focuses on technical aspects and test accuracy. Could this be put in the context of the adolescent population under study? Is this likely to be specific to this population or a generic issue? As this seems to be a broadly recognised issue does it need to be in this paper? Similarly the discussion of sample size and power isn’t something generic to this study, could this not be linked to the difficulties with school registers. What is your advice for overcoming these issues in the future? Is there a more reliable way to predict likely recruitment numbers or should you arithmetically correct any school estimates?

 The ethical clearance was suggested to take a long time – are there any data from other clinical trials done during this period to support this, or is this typical for the setting? If not what were the specific issue that slowed the process? Was the face to face meeting an unusual event or does this happen frequently?

I have read this submission. I believe that I have an appropriate level of expertise to confirm that it is of an acceptable scientific standard, however I have significant reservations, as outlined above.
